# Anti-Racism and Anti-Colonialism Praxis in Global Health—Reflection and Action for Practitioners in US Academic Medical Centers

**DOI:** 10.4269/ajtmh.21-0187

**Published:** 2021-07-19

**Authors:** Zeinabou Niamé Daffé, Yodeline Guillaume, Louise C. Ivers

**Affiliations:** 1 Center for Global Health, Massachusetts General Hospital, Boston, Massachusetts;; 2 Harvard Medical School, Boston, Massachusetts

## Abstract

The movement to decolonize global health and address power inequities among its actors is not new. Founded on the work of colonized and marginalized people themselves, initiatives at universities, schools of public health, and international development organizations have emerged to call for anti-racism and anti-colonialism within the field. US Academic Medical Centers (AMCs) have been less vocal in this wider discussion, despite their large engagement in the field through clinical, research, and medical education activities. As global health practitioners currently based at an AMC, we believe that it is important to critically evaluate our practices. We therefore propose three starting questions for our colleagues and students to consider and act upon as they adopt and navigate a praxis in anti-racism and anti-colonialism as foundational principles in global health. These questions call on us to closely examine the legacies of racism and colonialism in global health, the value placed on different ways of knowing in this field, and our motivations for engaging in this work. They are presented as a tool to reexamine global health, challenging the constructed binary of the “global South” and “global North,” and the perceived ideas of poverty and resource scarcity as the natural immutable reality of the global South.

## INTRODUCTION

Global health practitioners in academic medicine must reckon with certain realities of our work. These relate to the history of the field and its conception in colonialism as well as present-day inequities that continue to be pervasive.^1^^,^^2^ Such inequities include asymmetric power dynamics that often place institutions and practitioners in the “global North” above those in the “global South” and that normalize unequal partnerships where expertise and experience are more readily assigned to those of us in the global North. In the United States (where the authors are currently based), we, as global health practitioners, must understand and address our positionalities—the power and other differences in our social positions that shape our identities and access in society^3^—and the inherent power structures that exist as we strive to achieve shared goals of addressing the most pressing global health challenges of our time.

Academic Medical Centers (AMCs) play a unique role in the US global health landscape, falling at the intersection of clinical care, research, policy, and medical education, and their faculty and trainees engage in many global health partnerships—with increasing frequency.^4^ Despite this prominent role, we find that not all global health programs at AMCs have critically interrogated their position within the ill-defined field of “global health,”^5^ or what the term “partnership” does or should mean in the social, historical, and political context of global inequities. Yet all global health practitioners must be deliberate in how they engage in the work, question their practices, and actively work to be anti-racist and to “decolonize” global health programs, not only as an ideological but also a pragmatic commitment. The idea of decolonizing global health—identifying, unveiling, and interrupting the persisting remnants of colonialism within our practice and structures^6^—is not new.^7^^,^^8^ But AMCs have been institutionally less vocal in the wider discourse on this topic compared with universities, schools of public health, and even international development organizations.^9^ Less focus has been placed on “how” the global health work in AMCs is achieved—but we believe that the “how” really matters.^10^ AMCs must play a crucial role in this decolonizing work through institutional and individual efforts. In this article, we focus on individual efforts, proposing three initial questions as part of a continuous practice of self-reflection and inquiry for individuals who engage and design global health programs. These questions are intended to act as tools for global health practitioners at AMCs to unceasingly interrogate our practices and advocate for institutional change.

## HOW DO I UNDERSTAND THE LEGACIES OF RACISM AND COLONIALISM IN GLOBAL HEALTH?

Colonialism is an economic, social, and political system that relies on the principles of cultural hierarchy and supremacy as justification for the multifaceted domination of the “other.”^11^^,^^12^ The power structure of colonialism both relies on and perpetuates ideas of racial differences and superiority as tools for economic exploitation and cultural sovereignty—infantilizing colonized populations and situating them as incapable of self governance^13^—using evolutionary and essentialist rationalizations.^14^ In doing so, beyond its fundamental economic function,^15^ colonialism formalizes, institutionalizes, and structures racism.

The history of global health is inextricably linked to colonial conquest. The early interventions of what was then called international health were developed within colonial settings and to a large degree, were dependent on the coercive power of colonial rule.^16^^,^^17^ Shaped by colonial ideas that colonized peoples were incapable of improving their own health, the existence of local medical knowledge and existing public health infrastructures in colonized lands were subverted and colonial health services were designed primarily to protect the health of European and American personnel who were essential to upholding the colonial economy.^18^ This reality entangled colonial medicine with international health, shaping the development of the field. The emergence of schools of tropical medicine and hygiene in that epoch ensured that knowledge production in colonial settings was incorporated into the field of international health. Over time, these ideas and practices became the basis of expertise about health problems in the “developing world.”^19^ As a result, even in post- and non-colonial settings, international health experts continued to perpetuate colonial ideas that became “naturalized as global health science,”^19^ and it is this that we seek to disrupt.

Colonialism also creates what is known as colonial discourse—the system of statements and representations that historically characterized the colonized and the colonizer in binary, dualistic terms, and continues in the present.^20^ This binary classification of colonizer as the white civilized teacher (the “definer”), and the colonized as the black/brown primitive student (the “defined”) is one that global health has not yet sufficiently disrupted. It is also reflected in the limited and imprecise language used in global health to describe a “developing” world and one that is already “developed.” Other binary terms, such as global South/global North, resource-rich/resource-limited, cement this binary logic and lump vastly different communities, geographies, histories, and issues together, despite their heterogeneity and individual complexities. As one example, the vastly different regions of Africa, Asia, Latin America, and the Middle East are codified as homogenous, and often portrayed as immutably poor, and disease-ridden under the term “global South.” Moving beyond these constructs remains a challenge and we too are complicit in using these terms. It, however, remains important to reimagine these limiting classifications despite the challenges.

The effect of such colonial discourse is dangerous. It not only proliferates narratives surrounding race and ideas around needing to control and intervene in communities that are deemed incapable of doing so for themselves but also leads to detrimental actions. This is seen, for example, in short-term international trips for students and trainees that often raise several ethical concerns.^21^^,^^22^ Among these concerns, particularly relevant for AMCs, is medical tourism and the risk of trainees practicing beyond their skills without appropriate supervision.^21^ This is often coupled with limited actual contributions by AMCs to medical care delivery or health systems despite this being an area of their expertise, differentiating them from schools of public health. It is imperative for us as global health practitioners to understand these constructs, and to challenge the remnants of colonial discourse that still perpetuate in our institutions, partnerships, hypotheses, science, and writing, and to take actions to redress them.

## HOW DO I VALUE DIFFERENT WAYS OF KNOWING IN GLOBAL HEALTH?

Among the many acts of violence imposed on colonized populations, “epistemic violence”—the process by which entire sets of knowledge are disqualified as inadequate, naive, and lacking in science^23^—is an insidious form that continues to be reproduced in our field^24^ and to which AMCs may be particularly responsible for replicating—given their position as centers of knowledge generation, medical education, and dissemination. This violence underscores the presence of coloniality, and more specifically in this case, “coloniality of knowledge” or the structure of control whereby knowledge and ways of knowing is ascribed exclusively to colonial powers.^25^ This coloniality has contributed to the asymmetric power imbalances we see in regard to our dominant ways of knowing in global health.

Under colonialism, framing indigenous and other major knowledge systems as uncivilized and illegitimate is part of a racialized agenda to distinguish colonizers and colonial ways as inherently superior.^26^ This opposition process serves to undermine precolonial systems of knowledge and fix them as traditional, static, and outdated despite their dynamic nature and major contributions to innovations in every discipline including science.^26^ This racialized process of denying humanity to certain groups of people is coupled with denying them any virtue of knowing.^27^ For example, in many regions of the world there is a strong oral tradition as it relates to history and knowing,^28^ which like many precolonial ways of knowing, is often deprivileged and marginalized in the conventional process of knowledge production^29^ yet taken advantage of financially. This is reflected in “biopiracy”—the granting of patents to scientists who are not the holders of certain traditional knowledges.^30^^,^^31^ For instance, species in the *Phyllanthus* genus have been patented by researchers though these plants have been used for centuries in countries such as China and India for treating ailments including skin diseases and hypertension.^32^

In global health, one way the “coloniality of knowledge” shows up is in our norms and perceptions surrounding expertise. By knowingly or unwittingly devaluing the expertise of local collaborators and indigenous populations—their lived experience or other ways of knowing, we reproduce colonial practices, and miss critical opportunities for impact. In our global health practice, we believe that the knowledge of those most proximate to the problem at hand should be privileged, and that collective, inclusive problem-solving requires questioning academic perspectives on hierarchies of knowing. We must have the humility to recognize the possibility of plurality and diversity in knowledge^33^—to act on the idea that knowledge production exists beyond formal institutions and research experts^34^ and recognize the limits of our own contributions, and the gaps in our knowledge.

## WHAT ARE MY MOTIVATIONS FOR GLOBAL HEALTH?

Understanding the gross extraction of resources and human labor from colonized lands, and the dehumanization used to justify this extraction,^35^ we endorse the adoption of anti-racism as an explicit value in the global health field. This endorsement calls on us to interrogate our motivations for global health beyond the ideas of charity, aid, altruism, or wanting to “do good”—ideas that may be too reductionist, too accepting of the status quo of a “global North and global South” binary, and that normalize the deep economic injustices that have created global power and resource imbalances.^36^ Instead, by decentering ourselves in our motivations and reimagining global health as a work of global solidarity, we can engage in the idea of global health as global justice, a form of social empathy that relates to others in their efforts to overcome oppression and diminish suffering.^37^

Unlike aid or charity, solidarity goes beyond the notion of helping others and adopts a commitment to eliminating oppressive systems.^37^ Adopting a framework of justice, rooting our work in a common understanding of the histories of disenfranchisement that have created the disparities in power relations among nation states and between actors of global health is important. In doing so, we see resource imbalances and underdevelopment in certain regions of the world as direct vestiges of “capitalist, imperialist, and colonialist exploitation”^38^ as opposed to the natural order of the world. We conceive of global health as an ongoing commitment and a practice in which we commit ourselves to learning, unlearning, and relearning as a consistent process. This framework also calls for us as practitioners to constantly reflect on our social location and our positionalities, critically asking ourselves how our multiple intersecting identities inform and interact with our work and how we think of and navigate our social and cultural identities as tools for advancement.

As faculty and trainees at AMCs consider and make decisions about their role in global health work, from our viewpoint in US-based institutions we encourage other academic clinicians, researchers, students, and medical educators to reflect on history, on their understanding of hierarchies of knowledge, and on their own motivations for the work—as a start—and drawing from these, to commit to continuously improving their practice of anti-racism and anti-colonialism in global health through their actions. We welcome the conversations that ensue.
